# Supplementary material for: Lateral Propagation from Sensory Cortex Provides Differential Gain for Sensory Selection
Source: bioRxiv. 2026 Oct 28:2026.10.23.761374. Preprint. [Version 1] doi: 10.64898/2026.10.23.761374 (PMC13733777; doi:10.64898/2026.10.23.761374)
Supplement: Supplementary file 1 [file NIHPP2026.10.23.761374V1-supplement-1.pdf]

bioRxiv preprint doi: https://doi.org/10.64898/2026.10.23.761374; this version posted October 28, 2026. The copyright holder for this preprint (which was not certified by peer review) is the author/funder, who has granted bioRxiv a license to display the preprint in perpetuity. It is made available under a CC-BY 4.0 International license.

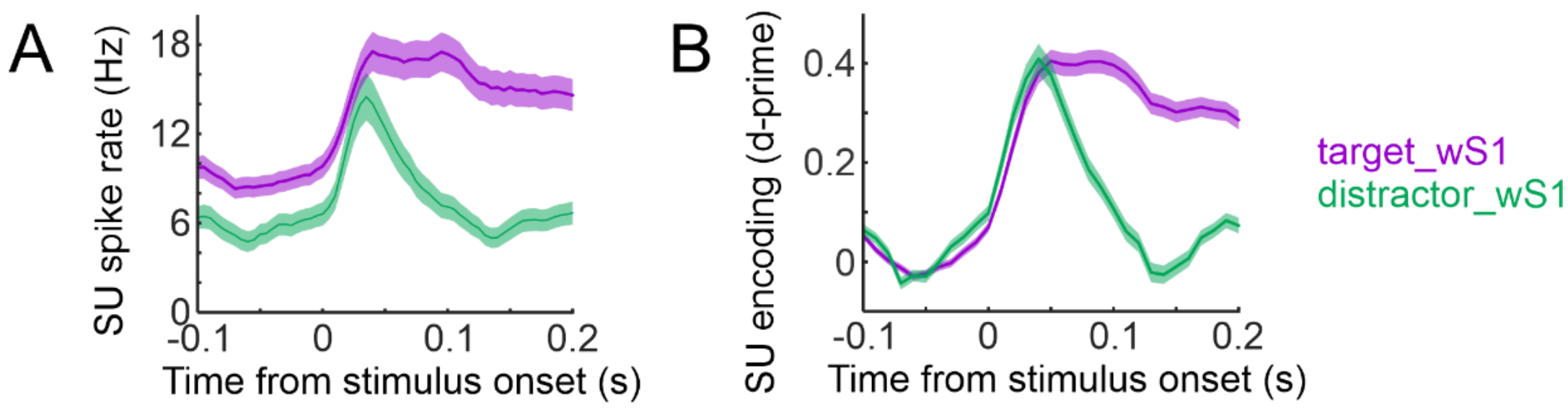

Supplemental Figure 1. Target and distractor stimuli evoke similar peak sensory encoding within their center response fields. (A) Mean single unit (SU) firing rates aligned to stimulus onset within the center response fields: target-aligned wS1 on target trials (target_wS1, purple) and distractor-aligned wS1 on distractor trials (distractor_wS1, green). (B) Neurometric stimulus encoding (d′) calculated from the same neuronal populations. Target and distractor populations exhibited similar rising phases and peak stimulus encoding. And yet, notable differences include higher prestimulus firing rates and sustained post-stimulus firing for the target compared to distractor population. Shading denotes SEM.

bioRxiv preprint doi: https://doi.org/10.64898/2026.10.23.761374; this version posted October 28, 2026. The copyright holder for this preprint (which was not certified by peer review) is the author/funder, who has granted bioRxiv a license to display the preprint in perpetuity. It is made available under a CC-BY 4.0 International license.

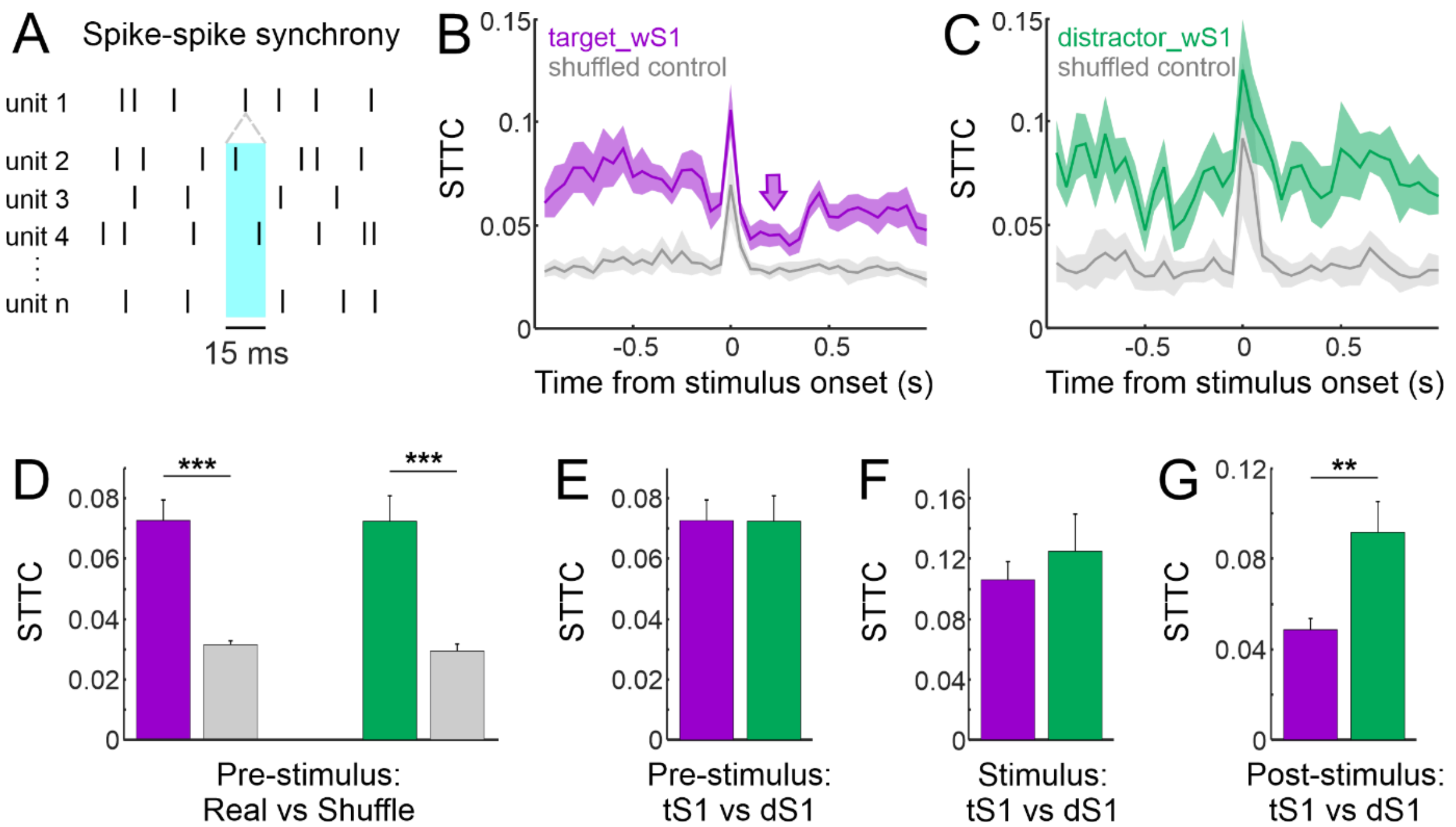

Supplemental Figure 2. Spike synchrony is not selectively enhanced for target stimuli in sensory cortex. (A) Illustration of spike synchrony, as the co-occurrence of spikes from pairs of neurons within a defined time window. (B,C) Spike Time Tiling Coefficient (STTC) results averaged across sessions, for the target_wS1 condition [B, purple], the distractor_wS1 condition [C, green], and their shuffled controls (gray). Both target_wS1 and distractor_wS1 exhibit similar prestimulus spike synchrony, which transiently increases at stimulus onset. Notably, target_wS1 displayed reduced spike synchrony post-stimulus (purple arrow in B). (D) STTC comparison of real data (color) and shuffled controls (gray) prestimulus (-1 second to stimulus onset). Both target_wS1 and distractor_wS1 exhibit spike synchrony significantly above chance levels. (E-G) STTC comparisons between target_wS1 and distractor_wS1. STTC distributions are not different prestimulus (E) or at stimulus onset (F, single window -50 ms to +50 ms). Post-stimulus (G, +50 ms to +200 ms), distractor_wS1 spike synchrony is greater than target_wS1

bioRxiv preprint doi: https://doi.org/10.64898/2026.10.23.761374; this version posted October 28, 2026. The copyright holder for this preprint (which was not certified by peer review) is the author/funder, who has granted bioRxiv a license to display the preprint in perpetuity. It is made available under a CC-BY 4.0 International license.

spike synchrony. **, p<0.01; ***, p<0.001. Error bars and shade are SEM, except for the shade of the shuffled controls which are 95% confidence intervals.

bioRxiv preprint doi: https://doi.org/10.64898/2026.10.23.761374; this version posted October 28, 2026. The copyright holder for this preprint (which was not certified by peer review) is the author/funder, who has granted bioRxiv a license to display the preprint in perpetuity. It is made available under a CC-BY 4.0 International license.

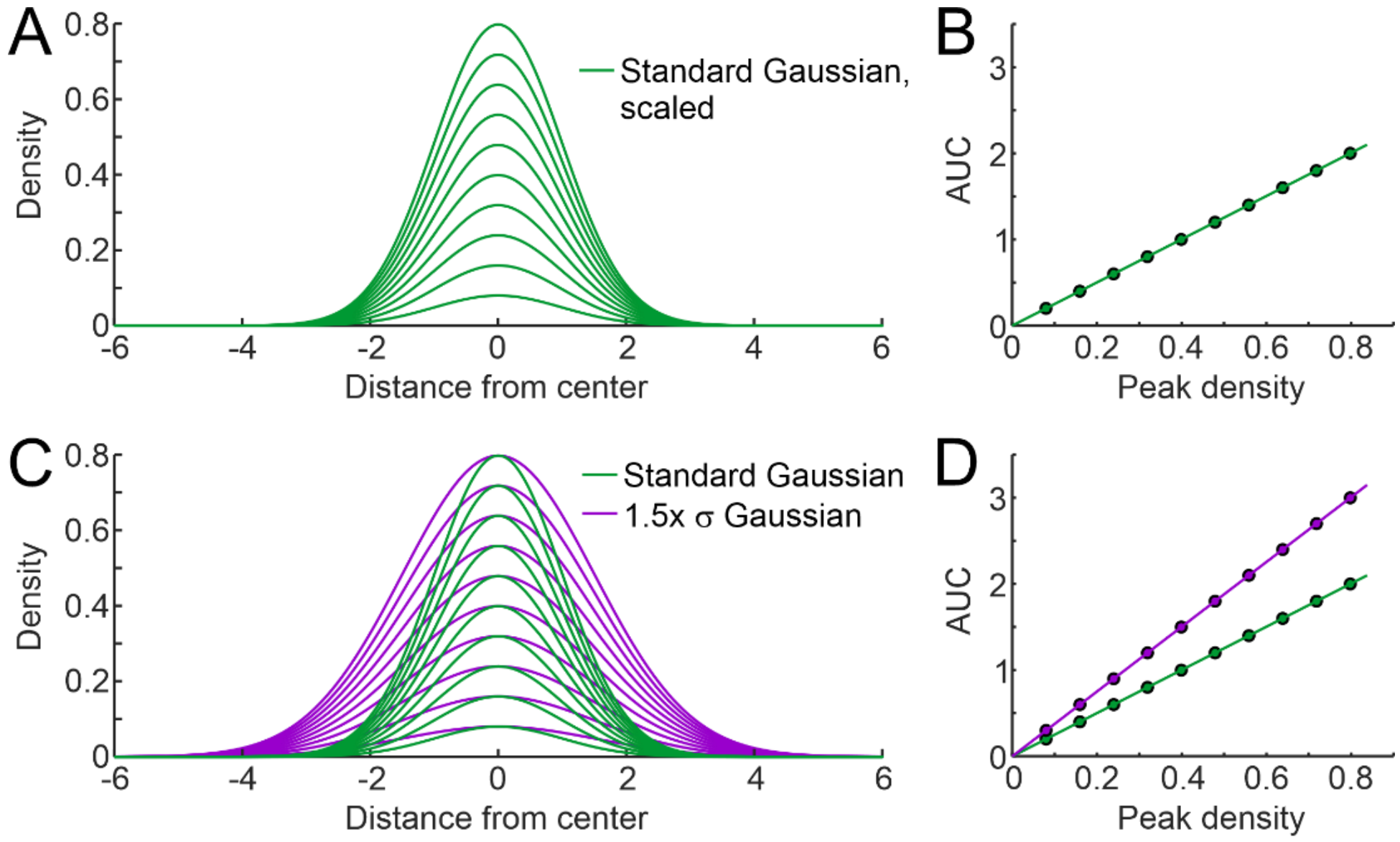

Supplemental Figure 3. Lateral gain increases the spatial spread of activity relative to peak response. (A) Simulation of gain as multiplicative scaling. Gaussian spatial activity profiles generated across a range of scales. (B) Integrative activity (Area under the curve (AUC), y-axis) plotted against peak activity (x-axis) demonstrates a preserved peak-spread relationship across multiplicative scales. (C) Simulation of gain as increases in the width relative to the standard distribution (purple). (D) Increasing distribution width produces a steeper relationship between integrated and peak activity. These simulations illustrate the different predictions of multiplicative scaling versus modulations of spatial propagation.

bioRxiv preprint doi: https://doi.org/10.64898/2026.10.23.761374; this version posted October 28, 2026. The copyright holder for this preprint (which was not certified by peer review) is the author/funder, who has granted bioRxiv a license to display the preprint in perpetuity. It is made available under a CC-BY 4.0 International license.

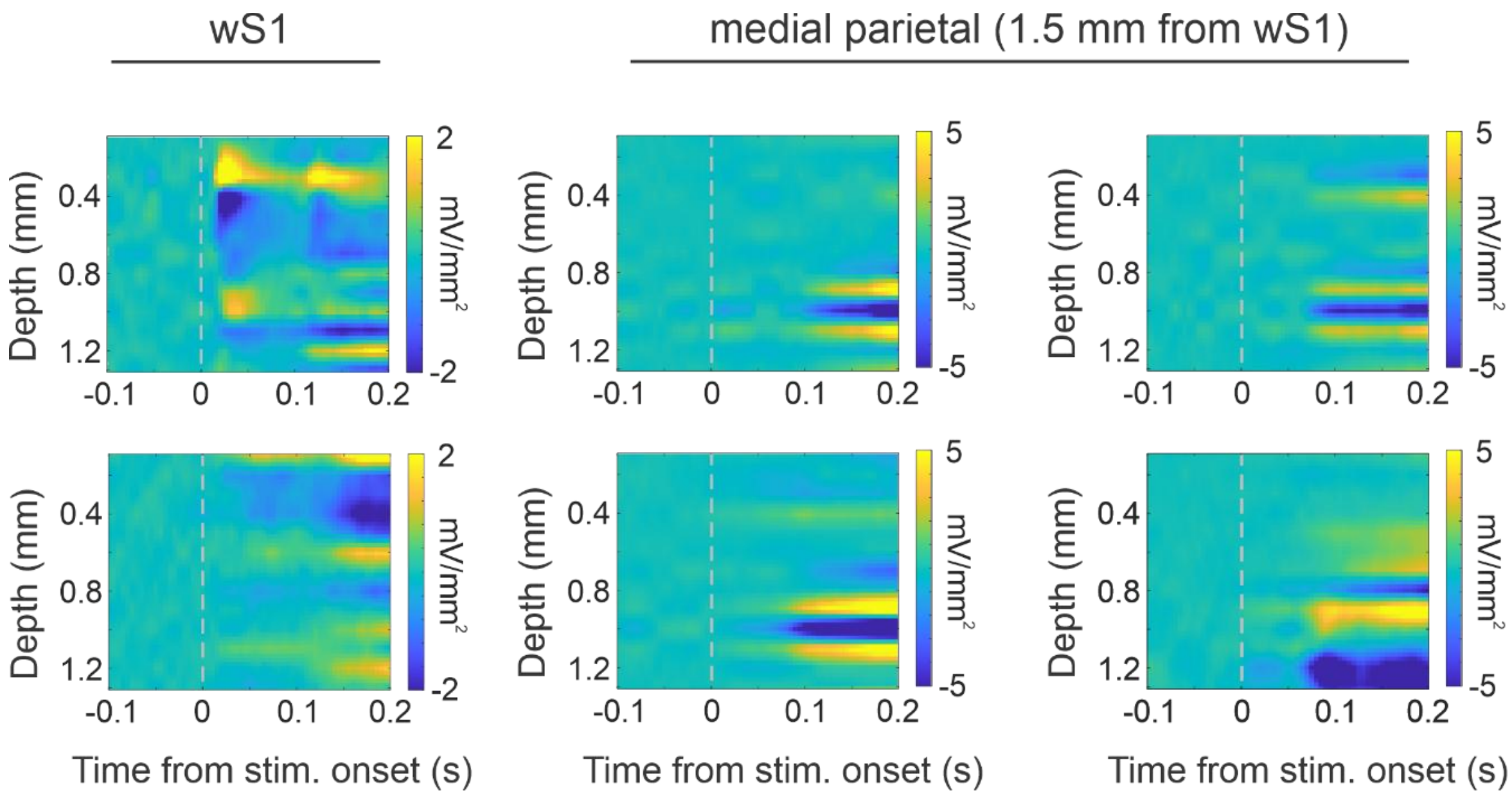

Supplemental Figure 4. Laminar current-source-density profiles of target-evoked responses within wS1 and medial parietal cortex. Representative CSD maps from independent recording sessions within wS1 (left column) and 1.5 mm medial to wS1 (middle and right columns). Stimulus onset is indicated by the vertical dashed line. wS1 recordings show prominent stimulus-evoked current sinks at intermediate cortical depths, whereas recordings medial to wS1 show prominent sinks in deeper cortical layers, consistent with preferential recruitment of infragranular layers during lateral propagation.

bioRxiv preprint doi: https://doi.org/10.64898/2026.10.23.761374; this version posted October 28, 2026. The copyright holder for this preprint (which was not certified by peer review) is the author/funder, who has granted bioRxiv a license to display the preprint in perpetuity. It is made available under a CC-BY 4.0 International license.

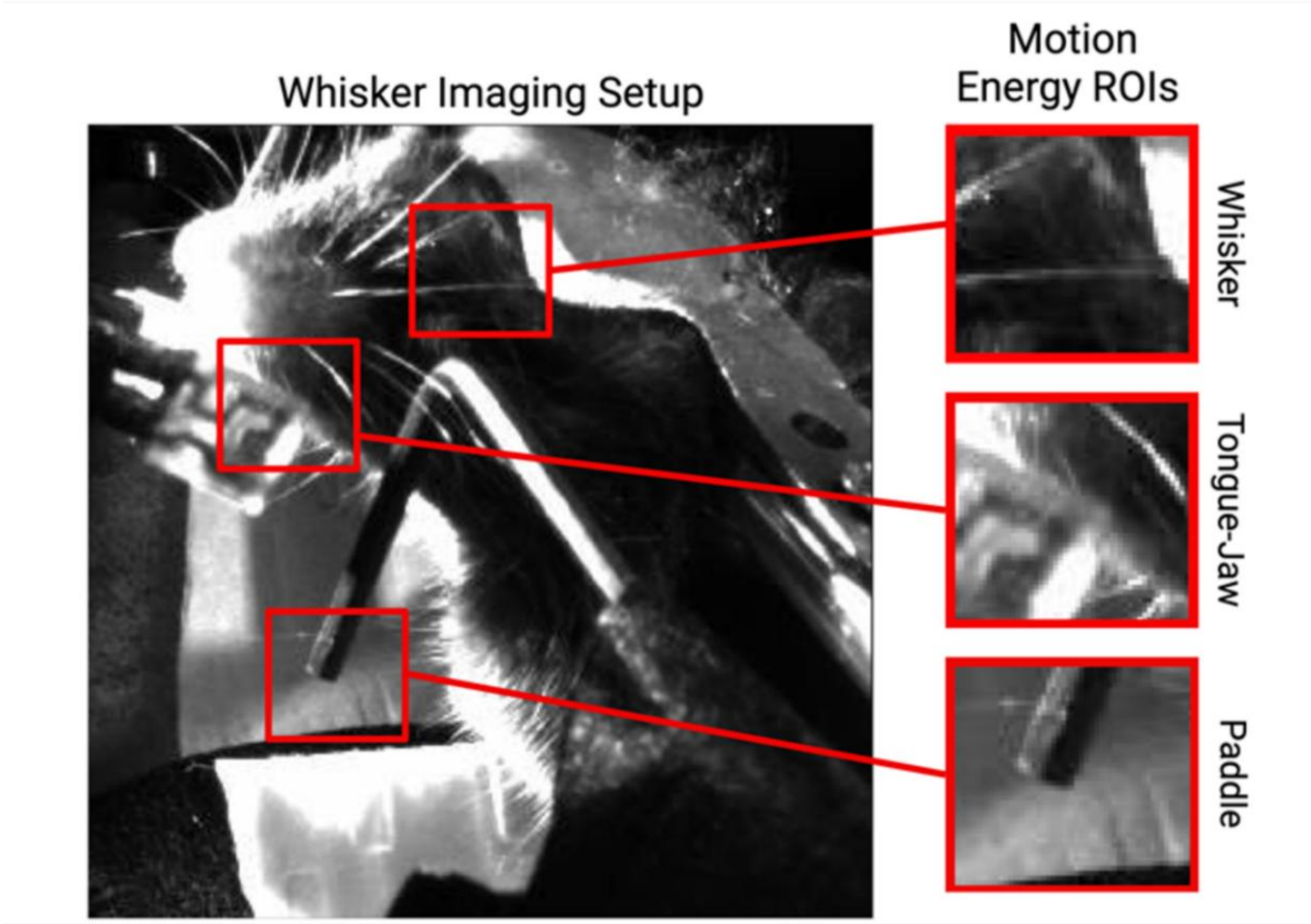

Supplemental Figure 5. Video-based regions of interest used for motion energy analyses. Representative behavioral video frame showing the head-fixed mouse, stimulus paddle, and face. Red boxes indicate regions of interest used to quantify motion energy from the unstimulated whiskers, tongue/jaw region, and stimulus paddle. Enlarged examples of each region are shown at right.

bioRxiv preprint doi: https://doi.org/10.64898/2026.10.23.761374; this version posted October 28, 2026. The copyright holder for this preprint (which was not certified by peer review) is the author/funder, who has granted bioRxiv a license to display the preprint in perpetuity. It is made available under a CC-BY 4.0 International license.

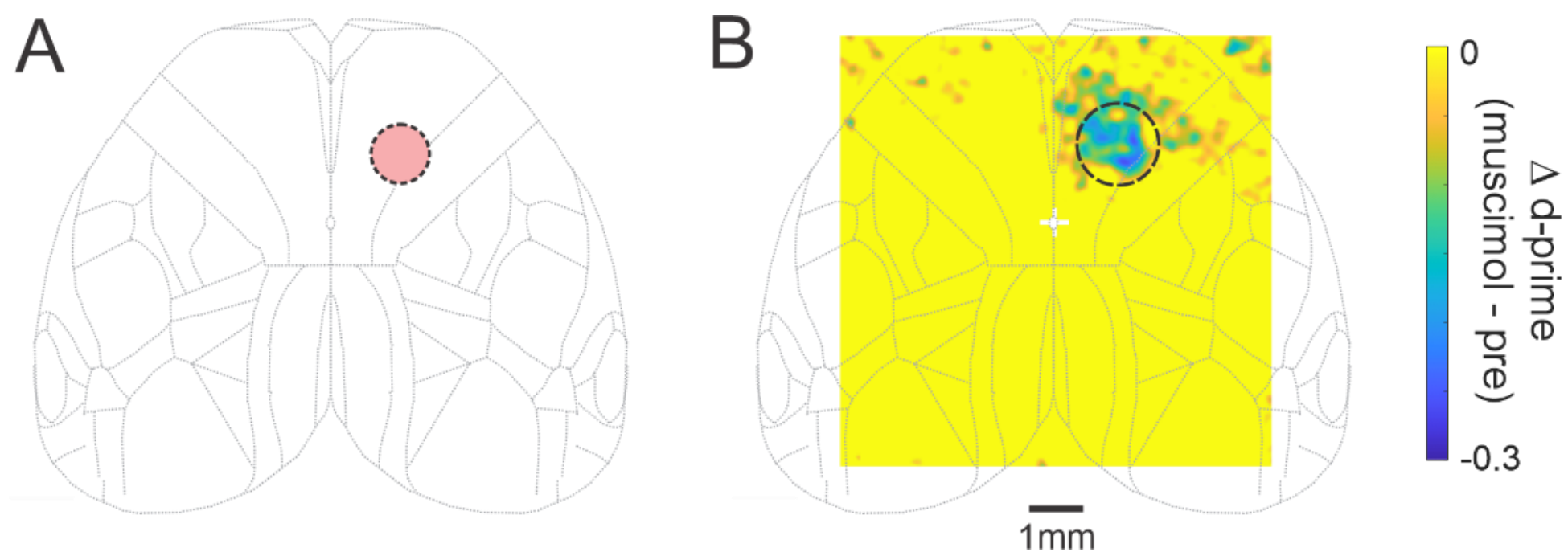

Supplemental Figure 6. Location and spatial extent of distractor-aligned whisker motor cortex suppression. (A) Schematic showing the location of muscimol injection into distractor-aligned whisker motor cortex (distractor_wMC). (B) Grand average difference map of cortical activity during the early response window (third post-stimulus frame), calculated as muscimol minus pre-muscimol activity. Negative values indicate reduced activity during muscimol suppression. Dashed circle indicates the approximate distractor_wMC injection region.

bioRxiv preprint doi: https://doi.org/10.64898/2026.10.23.761374; this version posted October 28, 2026. The copyright holder for this preprint (which was not certified by peer review) is the author/funder, who has granted bioRxiv a license to display the preprint in perpetuity. It is made available under a CC-BY 4.0 International license.

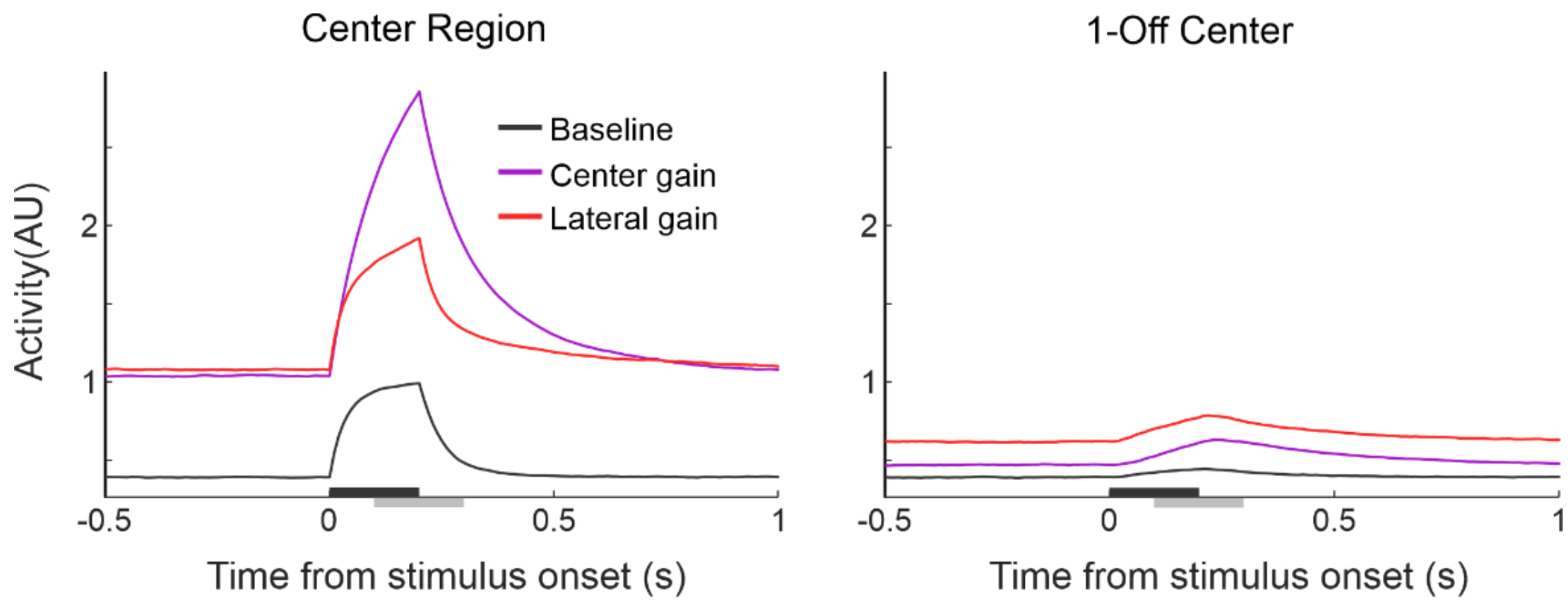

Supplemental Figure 7. Center and lateral gain differentially affect center and surround activity. Simulated activity over time within the center region (left) and an off-center region (right) under baseline, center gain, and lateral gain conditions. Increasing center gain strongly amplifies activity at the stimulated center while producing comparatively modest changes in the surrounding network. Increasing lateral coupling produces a disproportionate enhancement of off-center activity, illustrating how lateral gain can alter the spatial distribution of stimulus-evoked activity rather than simply scaling the center response. The black bar overlying the x-axis denotes the time of stimulus presentation; the gray bar denotes the post-stimulus analysis window.
